# A Survey of the Barriers Associated with Academic-based Cancer Research Commercialization

**DOI:** 10.1371/journal.pone.0072268

**Published:** 2013-08-21

**Authors:** Nathan L. Vanderford, L. Todd Weiss, Heidi L. Weiss

**Affiliations:** 1 Markey Cancer Center, University of Kentucky, Lexington, Kentucky, United States of America; 2 Business Division, Midway College, Midway, Kentucky, United States of America; 3 Department of Biostatistics, University of Kentucky, Lexington, Kentucky, United States of America; National Cancer Institute, National Institutes of Health, United States of America

## Abstract

Commercialization within the academic setting is associated with many challenges and barriers. Previous studies investigating these challenges/barriers have, in general, broadly focused on multiple disciplines and, oftentimes, several institutions simultaneously. The goal of the study presented here was to analyze a range of barriers that may be broadly associated with commercializing academic-based cancer research. This goal was addressed via a study of the barriers associated with cancer research commercialization at the University of Kentucky (UK). To this end, a research instrument in the form of an electronic survey was developed. General demographic information was collected on study participants and two research questions were addressed: 1) What are the general barriers inhibiting cancer research commercialization at UK? and 2) Would mitigation of the barriers potentially enhance faculty engagement in commercialization activities? Descriptive and statistical analysis of the data reveal that multiple barriers likely inhibit cancer research commercialization at UK with expense, time, infrastructure, and lack of industry partnerships being among the most commonly cited factors. The potential alleviation of these factors in addition to revised University policies/procedures, risk mitigation, more emphasis on commercialization by academia research field, and increased information on how to commercialize significantly correlated with the potential for increased commercialization activity. Furthermore, multivariate logistic regression modeling demonstrated that research commercialization would incrementally increase as barriers to the process are removed and that PhD-holding respondents and respondents in commercialization-supportive research fields would be more likely to commercialize their research upon barrier removal. Overall, as with other disciplines, these data suggest that for innovations derived from academic cancer-research to move more effectively and efficiently into the marketplace, university administrators and external agents, such as policymakers, need to address what are well-documented and defined issues.

## Introduction

The Bayh-Dole Act, which was passed by the United States Congress in 1980, gave academic institutions control over the intellectual property developed by faculty, staff, and/or students as the result of federally-sponsored research and it obligated institutions to license intellectual property with licensing preferences going to small businesses and industries within the United States. The Bayh-Dole Act also allowed universities and inventors to receive royalties from commercialized technology/services [1]–[4]. Prior to the Bayh-Dole Act, the United States government owned and managed intellectual property developed at academic institutions as the result of federal funds, and because of this arrangement, patent protection and licensing of technology was rarely pursued [1], [2]. As the result of advancements in academic- and industry-based research and changes in policies at the federal and institutional levels, academic institutions have become significant contributors to research commercialization via obtaining patents, licensing intellectual property, and forming start-up companies [5]. In fact, commercialization activity is increasing year-over-year. For example, in 2011, the Association of University Technology Managers indicated that surveyed institutions as a whole obtained 4,700 patents (increasing 5.2% over 2010), executed over 4,800 licenses (increasing 14% over 2010), and formed 670 start-up companies (increasing 3% over 2010). This activity earned the surveyed institutions $2.5 billion in income in 2011 representing a 2.6% increase over 2010 [6].

Despite a significant volume of academic-based research commercialization, academia faces challenges to commercializing innovations derived from its research. *C*hallenges that have been well-documented include: perceived risk; insufficient faculty time; lack of financial support; policy/regulation barriers; insufficient university commercialization infrastructure; lack of perceived importance to the university, the research field, to faculty, or to society; a disconnect between research and what faculty believe could be innovative, commercializable research; unclear and uncommon goals and benefits between faculty and university administration; faculty questioning whether research commercialization is a component of the academic mission; lack of entrepreneurial thinking among faculty; faculty not understanding how to commercialize their research; and lack of interaction and collaboration between universities and industry [7]–[14]. These challenges can reduce the effectiveness and efficiency of research commercialization resulting in academia-derived innovation that may never be commercialized to the market and thereby these challenges can hamper the downstream benefits that both academia and society could reap.

This study aimed to analyze a range of barriers that may be broadly associated with commercializing academic-based cancer research, which is an activity of interest to a number of faculty associated with the University of Kentucky (UK) Markey Cancer Center. This goal was addressed via a survey of the barriers associated with cancer research commercialization at UK. An electronic survey was developed and used to identify areas of opportunity for improving cancer research commercialization at UK. Two research questions were addressed: 1) What are the general barriers inhibiting cancer research commercialization at UK? and 2) Would mitigation of the barriers potentially enhance faculty engagement in commercialization activities? The data show that multiple barriers inhibit cancer research commercialization at UK. Additionally, multivariate logistic regression modeling demonstrates: 1) the potential for increasing cancer research commercialization at UK upon barrier removal; and 2) that some respondents are differentially impacted by the commercialization barriers. This analysis provides a roadmap for the issues that need to be addressed at UK to enhance cancer research commercialization. Additionally, given that the results are in-line with the existing data on the major challenges associated with academic-based research commercialization in general, these data further suggest that university administrators and external agents, such as policymakers, need to address a common set of issues to make all academic research commercialization more effective and efficient.

## Methodoloy

### Research Design

Similar previous studies that have broadly focused on identifying general research commercialization issues guided the development of the study presented here [7], [9], [14]. Of the prior research, the study herein is modeled most closely after the study conducted by the Atlantic Canada Opportunities Agency [7]. Inasmuch, the methodology and design of this study was quantitative, cross-sectional survey research.

A convenience sampling of UK faculty with interests in cancer research was used as study participants. As such, this is a cross-sectional study without a control population of researchers with broader research interests. At UK, the Colleges of Agriculture, Arts and Science, Communication and Information, Dentistry, Engineering, Health Sciences, Medicine, Nursing, Pharmacy, and Public Health house the majority of faculty members with potential cancer research interests. The selection criteria for inclusion in the study were that subjects must be UK faculty members with cancer-related research programs and/or support cancer research in some capacity. Subjects at the age of approximately 30 and above and, to the extent possible, of all genders, race, and ethnicity were recruited into the study. The recruitment population totaled 240 faculty, and 76 faculty participated in the study generating an overall response rate of 31.7%. The response rate for a particular question and/or responses to a particular variable within the research instrument did vary. For example, for questions related to research question 1, the response rate per barrier variable ranged from 28% to 31% (see Table S7), and for one aspect of research question 2, the response rate was 30% (see Table S9). Text S1 and Tables S1–S6 describe the demographic profile of the study participants.

The independent variables evaluating commercialization barriers and mitigating factors in this study were developed via the review of similar prior studies and included a selection of the various possible barriers/mitigating factors to commercializing academic research [7], [9], [14]. The measured independent variables fit into the themes of perceived risk; insufficient time; lack of financial support; policy/regulation barriers; insufficient university commercialization infrastructure; lack of perceived importance to the university, the research field, to faculty, or to society; faculty not understanding how to commercialize their research; lack of interaction and collaboration with industry; and the complexity of the research topic. Other measured variables included questions addressing the scope and importance of cancer research commercialization at UK in addition to other faculty demographic information such as age, race/ethnicity, academic department, academic rank, type of degree held, and type of research conducted.

### Instrumentation and Data Collection

As stated above, this study, including the data collection instrument, was modeled very closely after that of similar prior research with particular attention paid to the study by the Atlantic Canada Opportunities Agency [7]. The data collection/research instrument is included as supporting information (Text S2). Data were collected and managed using the Research Electronic Data Capture (REDCap) tool, which is available at UK. REDCap is a secure, Internet-based study-support application [15].

### Data Analysis

As indicated above, two research questions were addressed: 1) What are the general barriers inhibiting cancer research commercialization at UK? and 2) Would mitigation of the barriers potentially enhance faculty engagement in commercialization activities? The data instrumentation and collection methodology generated quantitative data which was aggregated for analysis.

A Likert scale was used for several of the questions within the research instrument. Descriptive statistics (frequencies and percent response) were calculated to initially summarize the distribution of the responses of the independent variables. The raw data (much of which is presented in the companion supporting information) was, in general, dichotomized (for example, yes or no; likely or not likely; agree or not agree) for further analysis. The Fisher’s exact test was used to explore the univariate association of barriers and mitigating factors with faculty’s commercialization engagement, namely whether faculty had attempted to commercialize their research (dichotomized as yes or no) or whether faculty would increase commercialization engagement if the barriers in the process were removed (dichotomized as agree [strongly agree, agree] or disagree [strongly disagree, disagree, neutral]). The barriers and mitigating factors were collapsed into two categories – namely agree (strongly agree, agree) or not agree (strongly disagree, disagree, neutral).

Multivariate analysis using the logistic regression model was employed to determine the effect of an overall cancer research commercialization barrier score and to understand whether some respondents are differentially impacted by different commercialization barriers. The overall barrier score was calculated based on the total number of barriers identified by each respondent and it produced a measure of the intensity of the barriers which was then included as an independent variable in the model. Additionally, the model measured the association of other independent variables – including age, gender, faculty rank, whether a respondent held a PhD versus MD, appointment in the College of Medicine, types of research a respondent is involved in, and the importance of commercialization in a respondent’s research field – with the intent to increase commercialization engagement if the barriers in the process were removed. Model-building using forward selection was employed and the final model included all the variables significantly associated with respondents’ intent to increase commercialization engagement upon barrier removal. The significant variables in the model are presented with odds ratios, p-values, and 95% confidence intervals.

### Ethical Considerations of Human Participants

The study design – including the informed consent process – of this human subjects research project was reviewed and approved by the University of Kentucky (UK) non-medical Institutional Review Board, which is administered through the UK Office of Research Integrity, a unit under the Office of the Vice President for Research. UK’s human research protection program is fully accredited by the Association for Accreditation of Human Research Protection Programs, Inc. Respondents were consented electronically via their engagement with the research instrument. Via REDCap, subjects remained both anonymous and confidential as no identifying codes were collected during the data collection stage of the study.

## Results

This cross-sectional study included responses from 76 faculty interested in cancer research at UK. The demographic profile of the study participants is described in the companion supporting text and tables (Text S1 and Tables S1–S6). Below, the results of the two research questions are summarized.

### Barriers Associated with Cancer Research Commercialization

**Figure 1:**
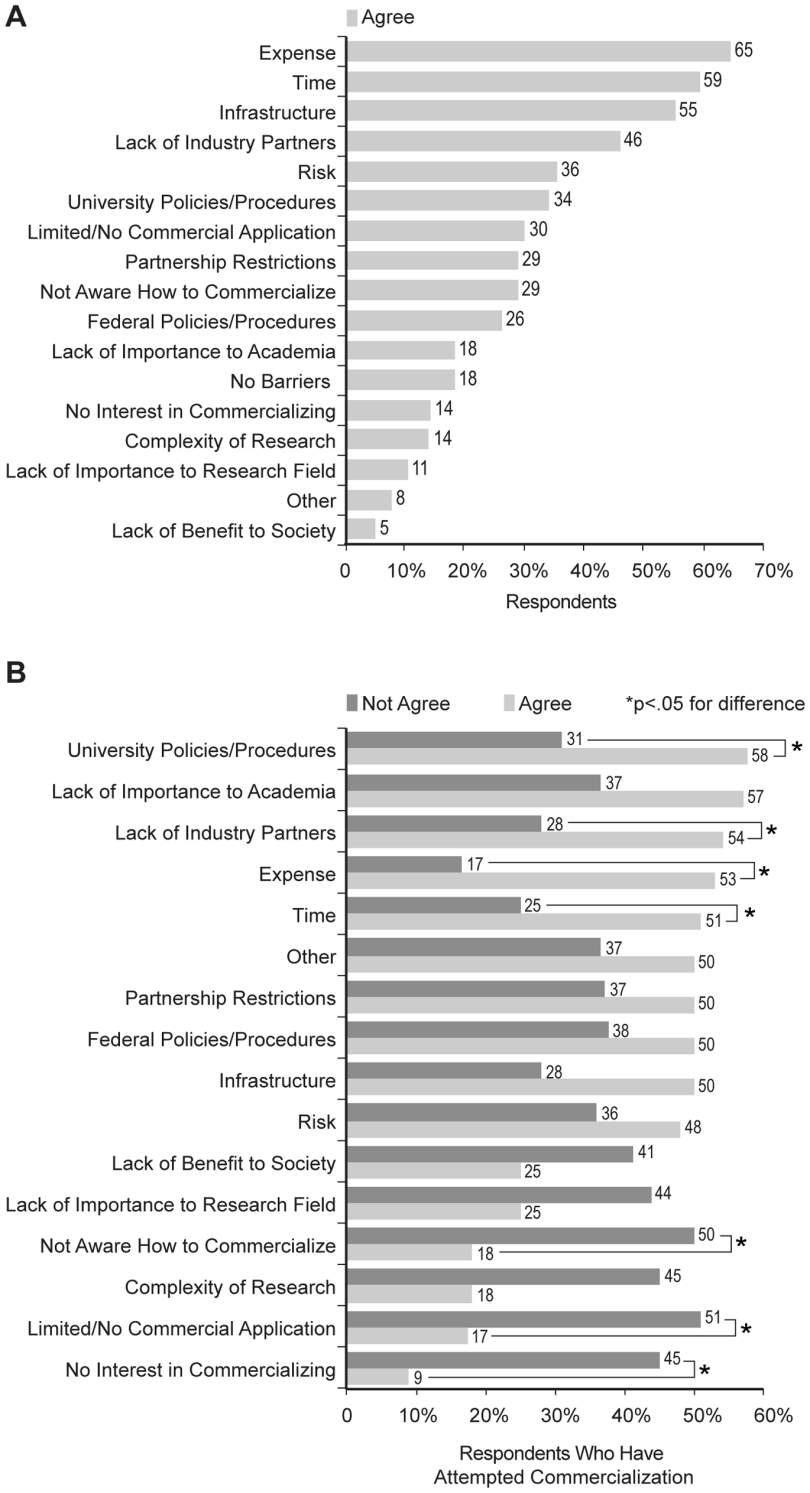
Barriers Associated with Cancer Research Commercialization. Respondents were asked to score whether a potential barrier (variable) is important to inhibiting their cancer research commercialization at UK. A) The percentage of respondents agreeing that a particular variable is a barrier to the commercialization of their cancer research. The raw data for panel A is shown in Table S7. B) The comparison (by percentage) of respondents indicating that they have attempted to commercialize their research and either agree versus not agree that a particular variable is a barrier to commercializing their research. The raw data for panel B is shown in Table S8. * p<0.05.

The first research question in this study was the following: What are the general barriers inhibiting cancer research commercialization at UK? To address this question, respondents were asked to score how important potential barriers are to inhibiting cancer research commercialization at UK. As shown in Figure 1A, expense (65%), time (59%), infrastructure (55%), and lack of industry partners (46%) were the most frequently chosen barriers that faculty felt inhibited their ability to commercialize their research. The Fisher’s exact test was utilized to measure potential association between the barriers and faculty’s engagement in the commercialization continuum via the attempt to commercialize their research (Table S4). Figure 1B shows that university policies/procedures (58% agree versus 31% not agree), lack of industry partnerships (54% agree versus 28% not agree), expense (53% agree versus 17% not agree), and time (51% agree versus 25% not agree) are significantly associated with faculty not attempting to commercialize their research. Also of note, not being aware how to commercialize (18% agree versus 50% not agree), limited research application (17% agree versus 51% not agree), and having no interest in commercializing (9% agree versus 45% not agree) do not – to a significant level – inhibit the respondents from attempting to commercialize their research.

### Mitigation Required to Increase Cancer Research Commercialization

**Figure 2:**
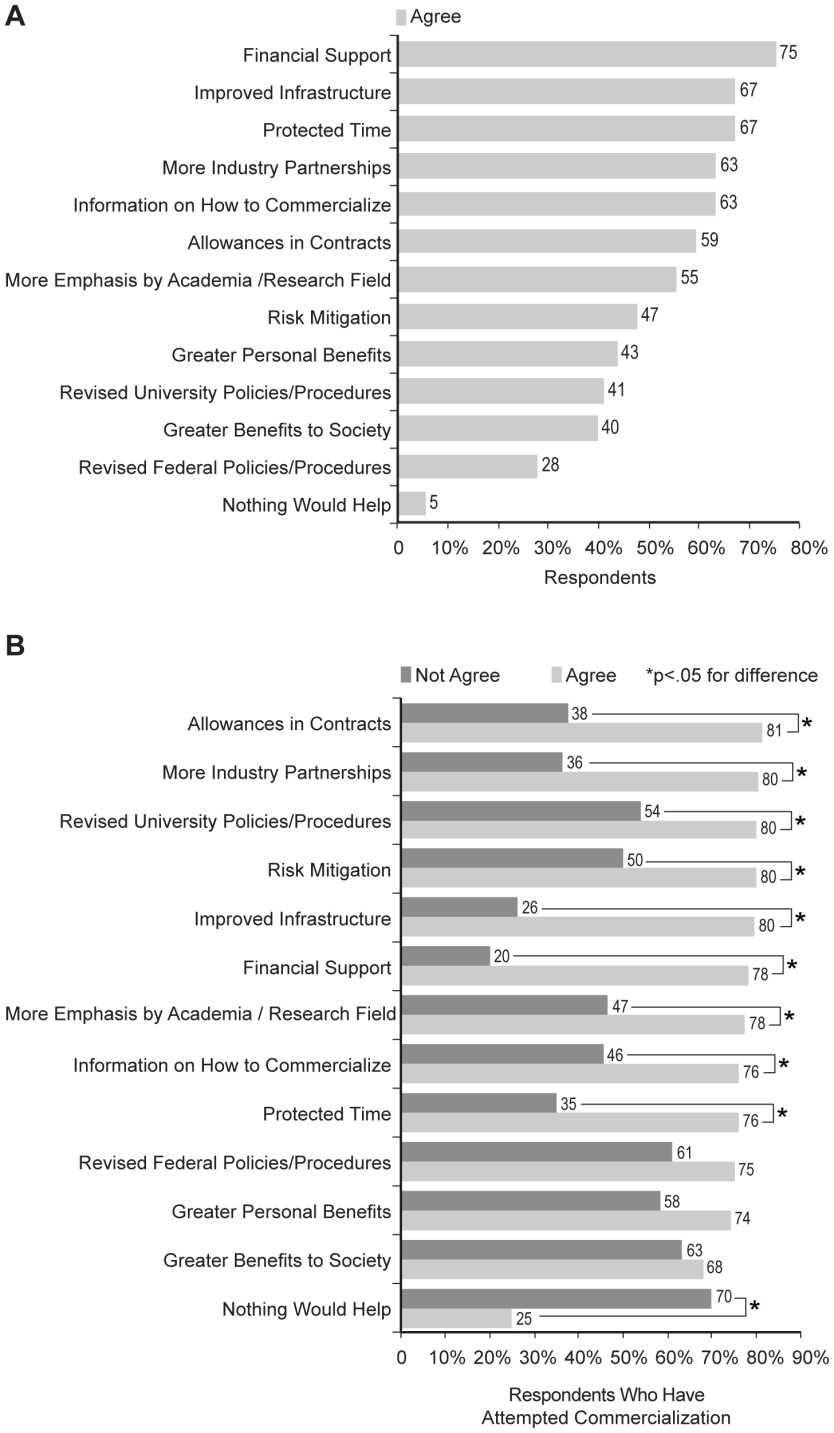
Mitigation Required to Increase Cancer Research Commercialization. Respondents were asked to indicate the mitigation that would be required to (theoretically) increase their participation in cancer research commercialization activities at UK. A) The percentage of respondents agreeing that a particular variable is a mitigating factor that would aid in increasing the commercialization of their cancer research. The raw data for panel A is shown in Table S10. B) The comparison (by percentage) of respondents indicating that they have attempted to commercialize their research and either agree versus not agree that a particular mitigating factor would aid in commercializing their research. The raw data for panel B is shown in Table S11. * p<0.05.

The second research question in this study was the following: Would mitigation of the barriers to research commercialization potentially enhance faculty engagement in commercialization activities? To address this question, respondents were simply asked if removing the barriers identified in research question 1 would increase their participation in research commercialization. As shown in Table S9, the data indicate that 61% of faculty believe that they would be more likely to participate in research commercialization if the barriers identified in research question 1 were removed. Next, using nearly identical variables as was used in research question 1, respondents were asked to indicate the mitigation that would be required to (theoretically) increase their participation in cancer research commercialization activities at UK. Figure 2A shows that financial support (75%), improved infrastructure (67%), protected time (67%), more industry partnerships (63%), information on how to commercialize (63%), allowances in industry partnership contracts (59%), and more emphasis by academia and/or a research field (55%) were the most frequently identified areas that faculty felt would need to be addressed to increase their research commercialization participation. The Fisher’s exact test was utilized to measure potential association between the mitigating factors and the potential for enhanced research commercialization. Figure 2B shows that allowances in industry contracts (81% agree versus 38% not agree), more industry partnerships (80% agree versus 36% not agree), revised university policies/procedures (80% agree versus 54% not agree), risk mitigation (80% agree versus 50% not agree), improved infrastructure (80% agree versus 26% not agree), financial support (78% agree versus 20% not agree), more emphasis placed on research commercialization by academia and/or a research field (78% agree versus 47% not agree), information on how to commercialize (76% agree versus 46% not agree), and protected time (76% agree versus 35% not agree) are significantly associated with faculty being more active in research commercialization. Also of note, acting somewhat as a control, respondents did not agree (25% agree versus 70% not agree) – to a significant extent – with the statement that “nothing would help” increase commercialization activity.

### Model of the Impact of Barrier Removal

Finally, a multivariate logistic regression model was used – as described in the Methodology section – to determine the effect of an overall cancer research commercialization barrier score and to understand whether some respondents are differentially impacted by different commercialization barriers. The calculated overall barrier score indicated that for every barrier mitigated, an individual would be 1.4 times more likely to agree to be more active in commercializing their research (p = 0.0008; 95% confidence interval = 1.1–1.6). The model also indicated that PhD-holding respondents (including PhDs and MD/PhDs) were 19 times more likely to agree to be more active in commercialization when the barriers in the process were removed versus respondents who did not hold this degree (p = 0.0004; 95% confidence interval = 3.7–97.7). And, respondents in research fields that are perceived to be supportive of academic research commercialization (Table S6) were found to be 5.5 times more likely to agree to be more active in commercialization of their research when the barriers were removed versus respondents in non-supportive research fields (p = 0.0219; 95% confidence interval = 1.3–23.8). The area under the receiver operating characteristic curve for this final model was approximately 89%, which indicates that the model correctly classifies about 89% of the sample into either one of the two levels of the outcome variable (agreeing to be more active in commercialization versus not agreeing to be so).

## Discussion

The challenges/barriers associated with academic-based research commercialization are well-documented and frequently studied. In very broad terms, the most frequently cited challenges/barriers are in the general categories of entrepreneurial history/culture of an institution, polices, infrastructure, and industry partnerships (7–14). The data presented in this current study are in-line with these previous observations, suggesting that the barriers to cancer research commercialization are no different than the barriers for other disciplines. As with other general studies, our data highlight the fact that no one barrier is likely solely responsible for inhibiting cancer research commercialization. Specifically, expense, time, infrastructure, and lack of industry partnerships are among the most frequently cited factors that our respondents felt inhibit their ability to commercialize their cancer research. Inasmuch, respondents indicated that they may be more active in commercialization if these factors in addition to University policies/procedures, risk, more emphasis by academia/a research field, and information on how to commercialize were alleviated/addressed. In fact, our multivariate logistic model demonstrated that multiple factors would indeed need mitigated to significantly boost cancer research commercialization as such activity would only increase by a factor of 1.4 for each barrier removed. Lastly, our model shows that PhD-holding faculty and faculty working in commercialization-supportive research fields are more likely to commercialize their research upon barrier removal than are other types of faculty.

It is important to note that several limitations are associated with this study. First, as a cross-sectional study, the findings may not be generalizable to all cancer-focused faculty either at UK or at other universities, and the findings may or may not be capable of being generalized to other research areas. Second, as a cross-sectional study, potential biases include subject selection bias which could lead to data and outcome bias. Additionally, since the study was designed to identify general challenges to cancer research commercialization, other, perhaps more specific, challenges may not have been addressed by this analysis. Lastly, the data was collected during a short period of time, and thus, barriers experienced by participants outside of this data collection widow may not have been captured. Despite these limitations, as stated above, the data herein are consistent with previous studies and therefore reasonable extrapolations can be made as to how these findings are applicable to UK and other institutions.

Many academic institutions have been successful at the commercialization process in ways that have led to increased commercialization activity and a greater volume of activity compared to what occurs at other universities. For example, in 2011, out of 186 respondents to the Association of University Technology Managers’ annual survey, 54 received more patents than the University of Kentucky (UK), 119 executed more licenses, 25 formed more start-up companies, and 90 generated more income from their commercialization activity than did UK [6]. Many of the highly commercializing institutions are being more successfully because they have navigated the barriers in the commercialization process in ways that facilitate enhanced commercialization activity [6], [8], [12]. As such, the data from this study can act as a roadmap for improving cancer-research commercialization at UK and perhaps at other institutions considering that our data are in-line with previous, general studies. For example, UK has 2 employees dedicated to intellectual property development whereas many other institutions have more staff dedicated to commercialization activities [6]. Such differences could be fairly easily reconciled, but these types of infrastructure-related issues are largely dictated by a university’s mission (or culture) regarding commercialization and the previous volume of commercialization activity within a university [8], [12]. The commercialization- and economic development-associated offices at UK have recently been organizationally restructured and the data from this study have been presented to the Vice President for Research, so these events may represent an opportunity to enhance the commercialization enterprise at the university.

Despite the fact that until now, this article has had an undertone of promoting an increase in academic-based research commercialization, this topic is under much international debate mostly over the ethical nature of such activity. The role of the university in the pursuit of general knowledge versus intellectual property protection and use thereof, general conflicts of interest potentially created by academic commercialization, and the role of profits from commercialization activities within the university setting are some of the issues central to the ethical debate [16]. Profits and incentives aimed at increasing research commercialization within academia have been argued to have the potential for stimulating the commercialization of “marginal inventions” and shifting universities from the pursuit of basic knowledge to the pursuit of only what has the greatest marketplace potential [17], [18].

Overall, the data herein adds to the current literature documenting the common and well-defined issues related to academic-based commercialization, as it appears that cancer research commercialization barriers are generally no different than those associated with other disciplines. As such, these data further outline the issues that university administrators and external agents, such as policymakers, need to address in order to move academic-derived research more effectively and efficiently into the marketplace. Notwithstanding, given the ethical issues related to academic-based research commercialization, perhaps some universities do not know how to devise commercialization practices and infrastructure in ways that will satisfy all the stakeholders and promote a healthy amount/balance of commercialization activity versus the general pursuit of knowledge. As such, prior to pursuing additional research in the area of the barriers/challenges associated with academic research commercialization, perhaps efforts in this field should shift to elucidating whether academic-based research commercialization is eroding or damaging the mission of academic research versus providing positive benefits to academia and society.

## Supporting Information

Table S1 Personal Demographics. (DOCX)

Table S2 Professional Demographics. (DOCX)

Table S3 Self-assessment of Professional Productivity. (DOCX)

Table S4 Research Commercialization Activity. (DOCX)

Table S5 Likelihood of Participating in the Three Areas of Research Commercialization. (DOCX)

Table S6 Importance of Participating in Research Commercialization. (DOCX)

Table S7 Data for Figure 1A (Challenges Associated with Cancer Research Commercialization). (DOCX)

Table S8 Data for Figure 1B (Association Between the Attempt to Commercialize and the Barriers To Commercializing). (DOCX)

Table S9 Impact of Removing Research Commercialization Barriers. (DOCX)

Table S10 Data for Figure 2A (Mitigation That Would Aid Increased Cancer Research Commercialization). (DOCX)

Table S11 Data for Figure 2B (Association Between Being More Active in Commercializing and Barrier Mitigation). (DOCX)

Text S1 Respondent Demographics. (DOCX)

Text S2 Research Instrument. (DOCX)
