# Supplementary material for: A Survey of the Barriers Associated with Academic-based Cancer Research Commercialization
Source: PLoS One. 2013 Aug 21;8(8):e72268. doi: 10.1371/journal.pone.0072268 (PMC3749229; doi:10.1371/journal.pone.0072268)
Supplement: Table S1 — (DOCX) [file pone.0072268.s001.docx]

| Table S1. Personal Demographics. | | |
| --- | --- | --- |
| Category | Subcategory | Frequency (Percent Response) |
| Gender | Male | 51(67.1) |
|  | Female | 20(26.3) |
|  | No Response | 5(6.6) |
| Age | Under 35 | 1(1.3) |
|  | 35-44 | 24(31.6) |
|  | 45-54 | 18(23.7) |
|  | 55-64 | 22(28.9) |
|  | 65-70 | 5(6.6) |
|  | 71 or older | 2(2.6) |
|  | No Response | 4(5.3) |
| Ethnicity/Race | American Indian or Alaska Native | 0 |
|  | Asian | 18(23.7) |
|  | Black or African American | 1(1.3) |
|  | Hispanic or Latino | 0 |
|  | Native Hawaiian or Other Pacific Islander | 0 |
|  | White | 51(67.1) |
|  | Other | 0 |
|  | No Response | 6(7.9) |
