# Supplementary material for: A Survey of the Barriers Associated with Academic-based Cancer Research Commercialization
Source: PLoS One. 2013 Aug 21;8(8):e72268. doi: 10.1371/journal.pone.0072268 (PMC3749229; doi:10.1371/journal.pone.0072268)
Supplement: Table S2 — (DOCX) [file pone.0072268.s002.docx]

| Table S2. Professional Demographics. | | |
| --- | --- | --- |
| Category | Subcategory | Frequency (Percent Response) |
| Academic Rank | Assistant Professor | 27(35.5) |
|  | Associate Professor | 18(23.7) |
|  | Professor | 30(39.5) |
|  | No Response | 1(1.3 |
| Degree | PhD (or equivalent) | 49(64.5) |
|  | MD (or equivalent) | 18(23.7) |
|  | MD/PhD | 9(11.8) |
|  | Other | 0 |
|  | No Response | 0 |
| College | College of Agriculture | 1(1.3) |
|  | College of Arts and Science | 3(3.9) |
|  | College of Communication and Information | 2(2.6) |
|  | College of Dentistry | 0 |
|  | College of Engineering | 2(2.6) |
|  | College of Health Sciences | 0 |
|  | College of Medicine | 56(73.7) |
|  | College of Nursing | 1(1.3) |
|  | College of Pharmacy | 6(7.9) |
|  | College of Public Health | 5(6.6) |
|  | Other | 0 |
|  | No Response | 0 |
| Research Category | Basic | 40(40) |
|  | Clinical | 22(22) |
|  | Translational | 26(26) |
|  | Population and/or Behavioral Science | 12(12) |
|  | No Response | 0 |
| Research Area | Drug Discovery, Delivery or Translational Therapy | 30(23.4) |
|  | Cell or Molecular Biology | 34(26.6) |
|  | Immunology | 8(6.3) |
|  | Tumor Microenvironment, Metastasis and/or Invasion | 14(10.9) |
|  | Cancer Prevention and Control | 25(19.5) |
|  | Biostatistics, Bioinformatics, or Informatics | 6(4.7) |
|  | Other | 11(8.6) |
|  | No Response | 0 |
