# Supplementary material for: A Survey of the Barriers Associated with Academic-based Cancer Research Commercialization
Source: PLoS One. 2013 Aug 21;8(8):e72268. doi: 10.1371/journal.pone.0072268 (PMC3749229; doi:10.1371/journal.pone.0072268)
Supplement: Table S3 — (DOCX) [file pone.0072268.s003.docx]

| Table S3. Self-assessment of Professional Productivity. | | |
| --- | --- | --- |
| Category | Subcategory | Frequency (Percent Response) |
| Satisfied with Level of Productivity | Yes | 52(68.4) |
|  | Neutral | 22(28.9) |
|  | No | 2(2.6) |
|  | No Response | 0 |
