# Supplementary material for: A Survey of the Barriers Associated with Academic-based Cancer Research Commercialization
Source: PLoS One. 2013 Aug 21;8(8):e72268. doi: 10.1371/journal.pone.0072268 (PMC3749229; doi:10.1371/journal.pone.0072268)
Supplement: Table S4 — (DOCX) [file pone.0072268.s004.docx]

| **Table S4.** Research Commercialization Activity. | | |
| --- | --- | --- |
| Category | Subcategory | Frequency (Percent Response) |
| Attempted to Commercialize | Yes | 30(39.5) |
|  | No | 45(59.2) |
|  | No Response | 1(1.3) |
| Successful at Commercializing | Yes | 24(31.6) |
|  | No | 49(64.5) |
|  | No Response | 3(3.9) |
| Intent to Commercialize in the Future | Yes | 40(52.6) |
|  | No | 34(44.7) |
|  | No Response | 2(2.6) |
