# Supplementary material for: A Survey of the Barriers Associated with Academic-based Cancer Research Commercialization
Source: PLoS One. 2013 Aug 21;8(8):e72268. doi: 10.1371/journal.pone.0072268 (PMC3749229; doi:10.1371/journal.pone.0072268)
Supplement: Table S5 — (DOCX) [file pone.0072268.s005.docx]

| Table S5. Likelihood of Participating in the Three Areas of Research Commercialization. | | | | | | |
| --- | --- | --- | --- | --- | --- | --- |
| Likert scale/Variable (Frequency [Percent Response]) | Highly Likely | Likely | Neutral | Unlikely | Highly Unlikely | No Response |
| Patenting | 21(27.6) | 17(22.4) | 15(19.7) | 10(13.2) | 12(15.8) | 1(1.3) |
| Licensing | 15(19.7) | 14(18.4) | 22(28.9) | 11(14.5) | 13(17.1) | 1(1.3) |
| Start-up Company Formation | 10(13.2) | 4(5.3) | 23(30.3) | 21(27.6) | 15(19.7) | 3(3.9) |
