# Supplementary material for: A Survey of the Barriers Associated with Academic-based Cancer Research Commercialization
Source: PLoS One. 2013 Aug 21;8(8):e72268. doi: 10.1371/journal.pone.0072268 (PMC3749229; doi:10.1371/journal.pone.0072268)
Supplement: Table S6 — (DOCX) [file pone.0072268.s006.docx]

| Table S6. Importance of Participating in Research Commercialization. | | | | | |
| --- | --- | --- | --- | --- | --- |
| Likert scale/Variable (Frequency [Percent Response]) | Very Important | Important | Neutral | Not Important | No Response |
| Importance in the Academic Setting | 26(34.2) | 28(36.8) | 19(25) | 1(1.3) | 2(2.6) |
| Importance to the Academic Mission | 7(9.2) | 16(21.1) | 34(44.7) | 18(23.7) | 1(1.3) |
| Importance to the Research Field | 7(9.2) | 29(38.2) | 23(30.3) | 17(22.4) | 0 |
