# Supplementary material for: A Survey of the Barriers Associated with Academic-based Cancer Research Commercialization
Source: PLoS One. 2013 Aug 21;8(8):e72268. doi: 10.1371/journal.pone.0072268 (PMC3749229; doi:10.1371/journal.pone.0072268)
Supplement: Table S7 — (DOCX) [file pone.0072268.s007.docx]

| Table S7. Data for Figure 1A (Challenges Associated with Cancer Research Commercialization). | | | | | | |  |
| --- | --- | --- | --- | --- | --- | --- | --- |
| Likert scale (score)/Variable (Frequency [Percent Response]) | Strongly Agree | Agree | Neutral | Disagree | Strongly Disagree | No Response | |
| No Barriers | 1(1.3) | 13(17.1) | 35(46.1) | 22(28.9) | 4(5.3) | 1(1.3) | |
| Risk | 3(3.9) | 24(31.6) | 41(53.9) | 5(6.6) | 1(1.3) | 2(2.6) | |
| Time | 10(13.2) | 35(46.1) | 25(32.9) | 3(3.9) | 0 | 3(3.9) | |
| Expense/Lack of Investors | 18(23.7) | 31(40.8) | 20(26.3) | 3(3.9) | 1(1.3) | 3(3.9) | |
| Infrastructure | 15(19.7) | 27(35.5) | 27(35.5) | 5(6.6) | 0 | 2(2.6) | |
| University Policies/Procedures | 10(13.2) | 16(21.1) | 38(50) | 8(10.5) | 1(1.3) | 3(3.9) | |
| Federal Policies/Procedures | 3(3.9) | 17(22.4) | 45(59.2) | 7(9.2) | 1(1.3) | 3(3.9) | |
| Lack of Industry Partners | 13(17.1) | 22(28.9) | 36(47.4) | 3(3.9) | 0 | 2(2.6) | |
| Partnership Restrictions | 6(7.9) | 16(21.1) | 43(56.6) | 7(9.2) | 1(1.3) | 3(3.9) | |
| Limited/No Commercial Application | 9(11.8) | 14(18.4) | 21(27.6) | 22(28.9) | 8(10.5) | 2(2.6) | |
| Complexity of Research | 3(3.9) | 8(10.5) | 38(50) | 18(23.7) | 6(7.9) | 3(3.9) | |
| Lack of Importance to Academia | 2(2.6) | 12(15.8) | 29(38.2) | 27(35.5) | 4(5.3) | 2(2.6) | |
| Lack of Importance to Research Field | 3(3.9) | 5(6.6) | 27(35.5) | 29(38.2) | 8(10.5) | 4(5.3) | |
| Lack of Benefit to Society | 1(1.3) | 3(3.9) | 24(31.6) | 28(36.8) | 18(23.7) | 2(2.6) | |
| Not Aware How to Commercialize | 8(10.5) | 14(18.4) | 29(38.2) | 13(17.1) | 8(10.5) | 4(5.3) | |
| No Interest in Commercializing | 2(2.6) | 9(11.8) | 24(31.6) | 23(30.3) | 17(22.4) | 1(1.3) | |
| Other | 1(1.3) | 5(6.6) | 48(63.2) | 5(6.6) | 7(9.2) | 10(13.2) | |
