# Supplementary material for: A Survey of the Barriers Associated with Academic-based Cancer Research Commercialization
Source: PLoS One. 2013 Aug 21;8(8):e72268. doi: 10.1371/journal.pone.0072268 (PMC3749229; doi:10.1371/journal.pone.0072268)
Supplement: Table S8 — (DOCX) [file pone.0072268.s008.docx]

| Table S8. Data for Figure 1B (Association Between the Attempt to Commercialize and the Barriers To Commercializing). | | | | | | |
| --- | --- | --- | --- | --- | --- | --- |
|  | | Have you ever attempted to commercialize your research (ie, submitted patent applications, attempted to licenses your research and/or attempted to create a start-up company, etc.)? | | | | p-value |
|  |  | Yes | | No | |  |
|  |  | Frequency Count | Percent of Row Frequency | Frequency Count | Percent of Row Frequency |  |
| Variable | Likert Level |  |  |  |  |  |
| Risk | Agree | 13 | 48.1 | 14 | 51.9 | 0.3360 |
|  | Not Agree | 17 | 36.1 | 30 | 63.8 |  |
| Time | Agree | 23 | 51.1 | 22 | 48.9 | 0.0312 |
|  | Not Agree | 7 | 25.0 | 21 | 75.0 |  |
| Expense and/or Lack of Investors | Agree | 26 | 53.1 | 23 | 46.9 | 0.0048 |
|  | Not Agree | 4 | 16.7 | 20 | 83.3 |  |
| Infrastructure | Agree | 21 | 50.0 | 21 | 50.0 | 0.0936 |
|  | Not Agree | 9 | 28.1 | 23 | 71.9 |  |
| University Policies/Procedures | Agree | 15 | 57.7 | 11 | 42.3 | 0.0468 |
|  | Not Agree | 15 | 31.1 | 32 | 68.1 |  |
| Federal Policies/Procedures | Agree | 10 | 50.0 | 10 | 50.0 | 0.4264 |
|  | Not Agree | 20 | 37.7 | 33 | 62.3 |  |
| Lack of Industry Partner | Agree | 19 | 54.3 | 16 | 45.7 | 0.0328 |
|  | Not Agree | 11 | 28.2 | 28 | 71.8 |  |
| Partnership Restrictions | Agree | 11 | 50.0 | 11 | 50.0 | 0.4372 |
|  | Not Agree | 19 | 37.3 | 32 | 62.8 |  |
| Limited or No Commercial Application of Research | Agree | 4 | 17.4 | 19 | 82.6 | 0.0098 |
|  | Not Agree | 26 | 51.0 | 25 | 49.0 |  |
| Complexity of Research | Agree | 2 | 18.2 | 9 | 81.8 | 0.1110 |
|  | Not Agree | 28 | 45.2 | 34 | 54.8 |  |
| Lack of Importance to Academia | Agree | 8 | 57.1 | 6 | 42.9 | 0.2275 |
|  | Not Agree | 22 | 36.7 | 38 | 63.3 |  |
| Lack of Importance to my Field | Agree | 2 | 25.0 | 6 | 75.0 | 0.4552 |
|  | Not Agree | 28 | 43.8 | 36 | 56.3 |  |
| Lack of Benefit to Society | Agree | 1 | 25.0 | 3 | 75.0 | 0.6424 |
|  | Not Agree | 29 | 41.4 | 41 | 58.6 |  |
| Not Aware How to Commercialize | Agree | 4 | 18.2 | 18 | 81.8 | 0.0179 |
|  | Not Agree | 25 | 50.0 | 25 | 50.0 |  |
| No Interest in Commercializing | Agree | 1 | 9.1 | 10 | 90.9 | 0.0417 |
|  | Not Agree | 29 | 45.1 | 35 | 54.7 |  |
| Other | Agree | 3 | 50.0 | 3 | 50.0 | 0.6656 |
|  | Not Agree | 22 | 36.7 | 38 | 63.3 |  |
