# Supplementary material for: A Survey of the Barriers Associated with Academic-based Cancer Research Commercialization
Source: PLoS One. 2013 Aug 21;8(8):e72268. doi: 10.1371/journal.pone.0072268 (PMC3749229; doi:10.1371/journal.pone.0072268)
Supplement: Table S9 — (DOCX) [file pone.0072268.s009.docx]

| Table S9. Impact of Removing Research Commercialization Barriers. | | | | | | |
| --- | --- | --- | --- | --- | --- | --- |
| Likert scale/Variable (Frequency [Percent Response]) | Strongly Agree | Agree | Neutral | Disagree | Strongly Disagree | No Response |
| Removal of Barriers Would Increase Commercialization Participation | 19(25) | 27(35.5) | 20(26.3) | 4(5.3) | 2(2.6) | 4(5.3) |
