# Supplementary material for: A Survey of the Barriers Associated with Academic-based Cancer Research Commercialization
Source: PLoS One. 2013 Aug 21;8(8):e72268. doi: 10.1371/journal.pone.0072268 (PMC3749229; doi:10.1371/journal.pone.0072268)
Supplement: Table S10 — (DOCX) [file pone.0072268.s010.docx]

| Table S10. Data for Figure 2A (Mitigation That Would Aid Increased Cancer Research Commercialization). | | | | | | |
| --- | --- | --- | --- | --- | --- | --- |
| Likert scale (score)/Variable (Frequency [Percent Response]) | Strongly Agree | Agree | Neutral | Disagree | Strongly Disagree | No Response |
| Risk Mitigation | 6(7.9) | 30(39.5) | 35(46.1) | 1(1.3) | 0 | 4(5.3) |
| Protected Time | 13(17.1) | 38(50) | 19(25) | 3(3.9) | 0 | 3(3.9) |
| Financial Support | 26(34.2) | 31(40.8) | 15(19.7) | 1(1.3) | 0 | 3(3.9) |
| Improved Infrastructure | 23(30.3) | 28(36.8) | 18(23.7) | 2(2.6) | 0 | 5(6.6) |
| Revised University Policies/Procedures | 15(19.7) | 16(21.1) | 38(50) | 3(3.9) | 0 | 4(5.3) |
| Revised Federal Policies/Procedures | 6(7.9) | 15(19.7) | 44(57.9) | 4(5.3) | 3(3.9) | 4(5.3) |
| More Industry Partnerships | 18(23.7) | 30(39.5) | 22(28.9) | 1(1.3) | 0 | 5(6.6) |
| Allowances in Contracts | 16(21.1) | 29(38.2) | 24(31.6) | 1(1.3) | 0 | 6(7.9) |
| More Emphasis by Academia and/or Research Field | 13(17.1) | 29(38.2) | 24(31.6) | 7(9.2) | 0 | 3(3.9) |
| Greater Benefits to Society | 7(9.2) | 23(30.3) | 29(38.2) | 11(14.5) | 2(2.6) | 4(5.3) |
| Greater Personal Benefits | 9(11.8) | 24(31.6) | 27(35.5) | 7(9.2) | 3(3.9) | 6(7.9) |
| Information on How to Commercialize | 15(19.7) | 33(43.4) | 21(27.6) | 1(1.3) | 1(1.3) | 5(6.6) |
| Nothing Would Help | 1(1.3) | 3(3.9) | 26(34.2) | 17(22.4) | 20(26.3) | 9(11.8) |
