# Supplementary material for: A Survey of the Barriers Associated with Academic-based Cancer Research Commercialization
Source: PLoS One. 2013 Aug 21;8(8):e72268. doi: 10.1371/journal.pone.0072268 (PMC3749229; doi:10.1371/journal.pone.0072268)
Supplement: Table S11 — (DOCX) [file pone.0072268.s011.docx]

| Table S11. Data for Figure 2B (Association Between Being More Active in Commercializing and Barrier Mitigation). | | | | | | |
| --- | --- | --- | --- | --- | --- | --- |
|  | | If the barriers you identified above were eliminated, would you be more active in commercializing your research? | | | | p-value |
|  |  | Agree | | Disagree | |  |
|  |  | Frequency Count | Percent of Row Frequency | Frequency Count | Percent of Row Frequency |  |
| Variable Label | Likert Level |  |  |  |  |  |
| Risk Mitigation | Agree | 28 | 80.0 | 7 | 20.0 | 0.0119 |
|  | Not Agree | 17 | 50.0 | 17 | 50.0 |  |
| Protected Time | Agree | 38 | 76.0 | 12 | 24.0 | 0.0021 |
|  | Not Agree | 7 | 35.0 | 13 | 65.0 |  |
| Financial Support | Agree | 43 | 78.2 | 12 | 21.8 | 0.0001 |
|  | Not Agree | 3 | 20.0 | 12 | 80.0 |  |
| Improved Infrastructure | Agree | 39 | 79.6 | 10 | 20.4 | 0.0001 |
|  | Not Agree | 5 | 26.3 | 14 | 73.7 |  |
| Revised University Policies/Procedures | Agree | 24 | 80.0 | 6 | 20.0 | 0.0403 |
|  | Not Agree | 21 | 53.9 | 18 | 46.2 |  |
| Revised Federal Policies/Procedures | Agree | 15 | 75.0 | 5 | 25.0 | 0.4043 |
|  | Not Agree | 30 | 61.2 | 19 | 38.8 |  |
| More Industry Partnerships | Agree | 37 | 80.4 | 9 | 19.6 | 0.0007 |
|  | Not Agree | 8 | 36.4 | 14 | 63.6 |  |
| Allowances in Contracts | Agree | 35 | 81.4 | 8 | 18.6 | 0.0005 |
|  | Not Agree | 9 | 37.5 | 15 | 62.5 |  |
| More Emphasis by Academia and/or my Research Field | Agree | 31 | 77.5 | 9 | 22.5 | 0.0115 |
|  | Not Agree | 14 | 46.7 | 16 | 53.3 |  |
| Greater Benefits to Society | Agree | 19 | 67.9 | 9 | 32.1 | 0.7994 |
|  | Not Agree | 26 | 63.4 | 15 | 36.6 |  |
| Greater Personal Benefits | Agree | 23 | 74.2 | 8 | 25.8 | 0.2044 |
|  | Not Agree | 21 | 58.3 | 15 | 41.7 |  |
| Information on How to Commercialize | Agree | 35 | 76.1 | 11 | 23.9 | 0.0160 |
|  | Not Agree | 10 | 45.5 | 12 | 54.6 |  |
| Nothing Would Help | Agree | 1 | 25.0 | 3 | 75.0 | 0.0994 |
|  | Not Agree | 42 | 70.0 | 18 | 30.0 |  |
