# Supplementary material for: A Survey of the Barriers Associated with Academic-based Cancer Research Commercialization
Source: PLoS One. 2013 Aug 21;8(8):e72268. doi: 10.1371/journal.pone.0072268 (PMC3749229; doi:10.1371/journal.pone.0072268)
Supplement: Text S1 — (DOCX) [file pone.0072268.s012.docx]

**Text S1**

Respondent Demographics

Out of the targeted 240 faculty at UK who perform or are involved in cancer-related research, seventy-six (76) participated in the study generating a response rate of 31.7%. The raw data describing the 76 respondents are shown in Tables S1-S6.

Table S1 shows the personal demographic profile of the respondents. Sixty-seven percent (67.1%) of the respondents were male and 26.3% were female. Eighty-four percent (84.2%) of the respondents were in the age range of 35-64. Sixty-seven percent (67.1%) of the respondents were White, 23.7% were Asian, and 1.3% were Black/African American. Table S2 shows the professional demographic profile of the respondents. Thirty-five and a half percent (35.5%), 23.7%, and 39.5% of respondents had the academic rank of Assistant, Associate, or Full Professor, respectively. Sixty-four and a half percent (64.5%) of respondents held a PhD or equivalent degree, 23.7% held an MD or equivalent degree, and 11.8% held a dual MD/PhD degree. Nearly 74% of respondents held faculty appointments in the College of Medicine, 7.9% were faculty in the College of Pharmacy, 6.6% were faculty in the College of Public Health, and the rest of the respondents held appointments in 5 other Colleges. Basic science research is the most highly represented (40%) research category among respondents while translational (26%), clinical (22%), and population and/or behavioral science (12%) was identified as the next most highly represented research categories. Within these research categories, respondents conduct research in a variety of areas with cell or molecular biology (26.6%) and drug discovery, delivery or translation therapy (23.4%) being the most common areas.

A series of questions were asked to characterize the scope and importance of cancer research commercialization among the respondents and the results are shown in Tables S3-S6. Table S3 indicates that nearly 70% of respondents felt satisfied with their level of professional productivity in terms of publishing research manuscripts, obtaining grant funding, and other means of productivity. Despite this, Table S4 shows that 59.2% of respondents had never attempted to commercialize their research while 39.5% had. Of those attempting to commercialize their research, 31.6% had been successful, and nearly 53% of respondents indicated that they intend to attempt to commercialize their research at some point in the future. Table S5 shows that 50% of the responding faculty indicated that they intend to participate in patenting activities, 38.1% of faculty intend to participate in licensing activities, and 47.3% indicated that they would not participate in start-up company formation. Lastly, respondents were asked to indicate the level of importance of research commercialization in the academic setting, to the academic mission, and to their research field of study. As shown in Table S6, the data indicate that 71% of faculty believe that research commercialization is important in the academic setting, only 30.3% of the faculty feel that research commercialization is important to the academic mission, and 47.1% of the faculty believe that their research field values research commercialization.
