# Supplementary figures and images for: A Survey of the Barriers Associated with Academic-based Cancer Research Commercialization
Source: PLoS One. 2013 Aug 21;8(8):e72268. doi: 10.1371/journal.pone.0072268 (PMC3749229; doi:10.1371/journal.pone.0072268)

**Text S2**

Research Instrument


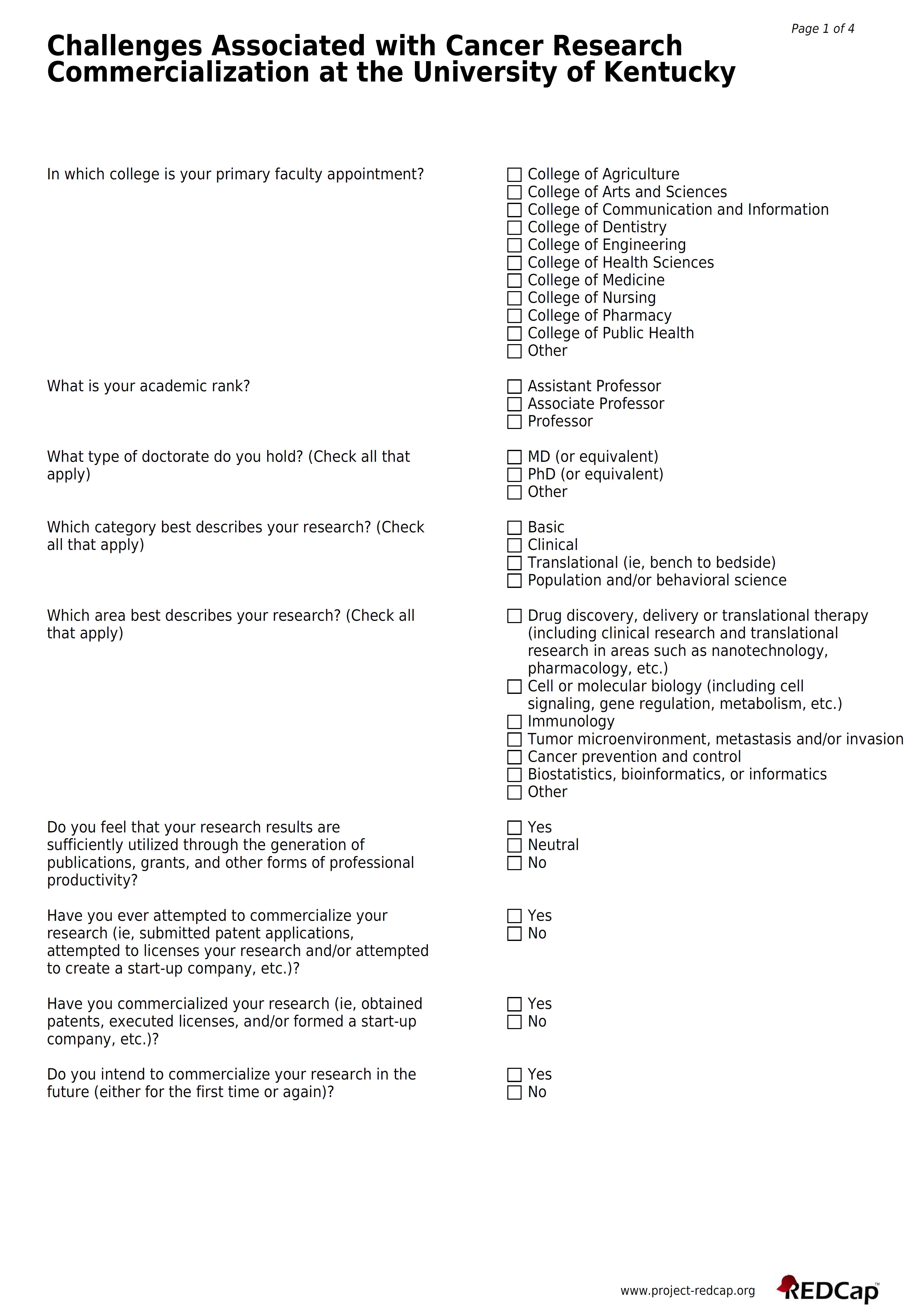


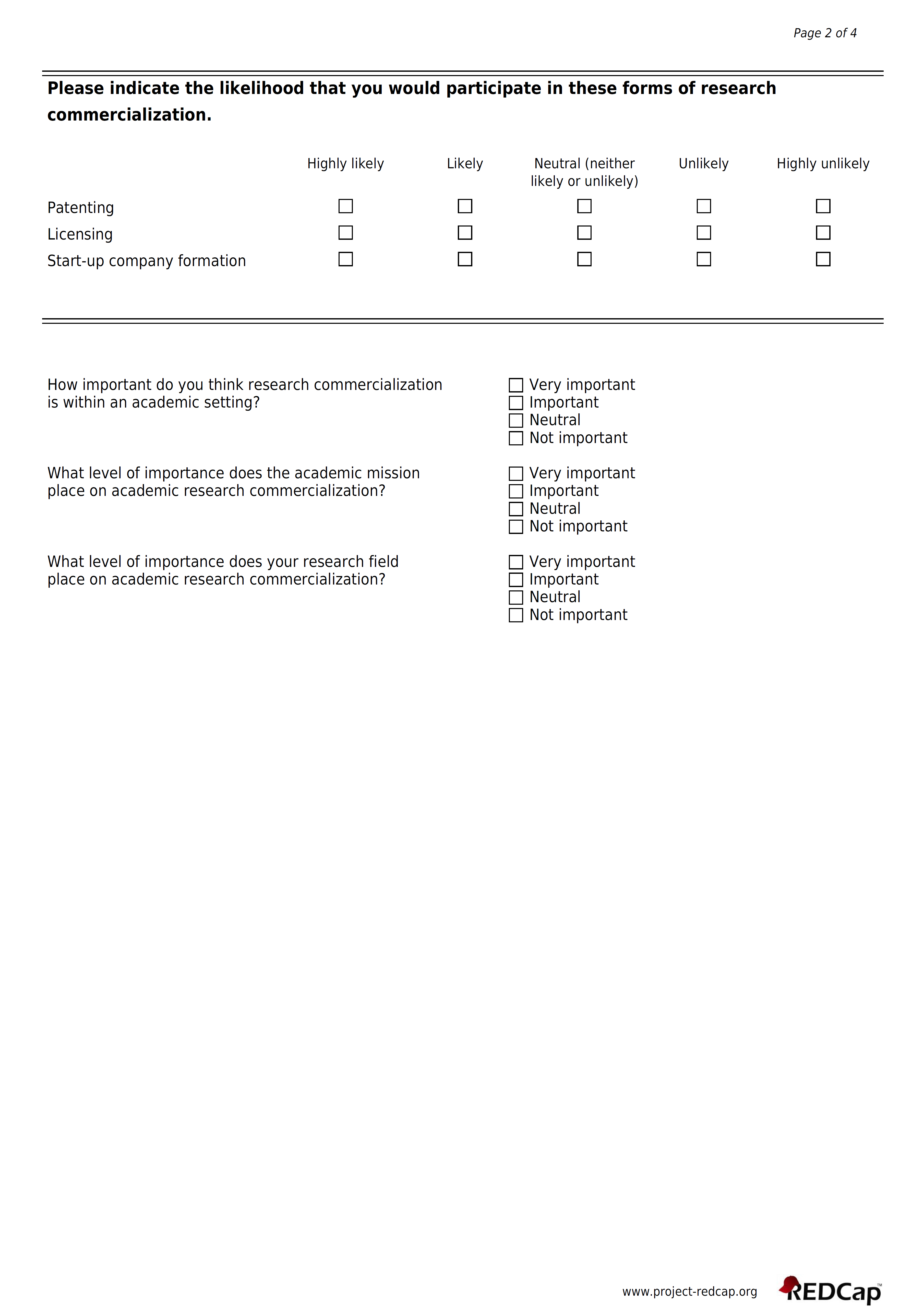


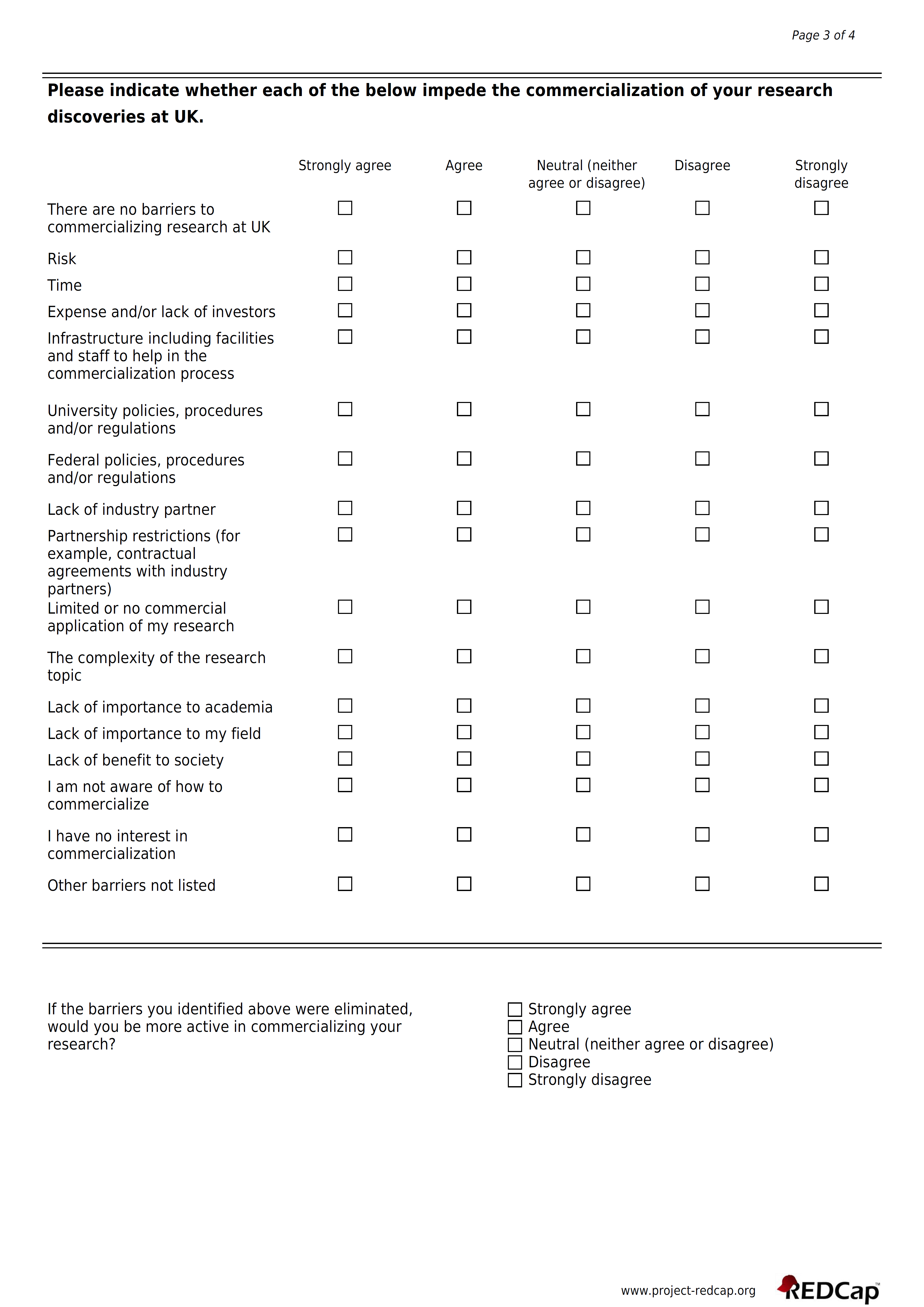


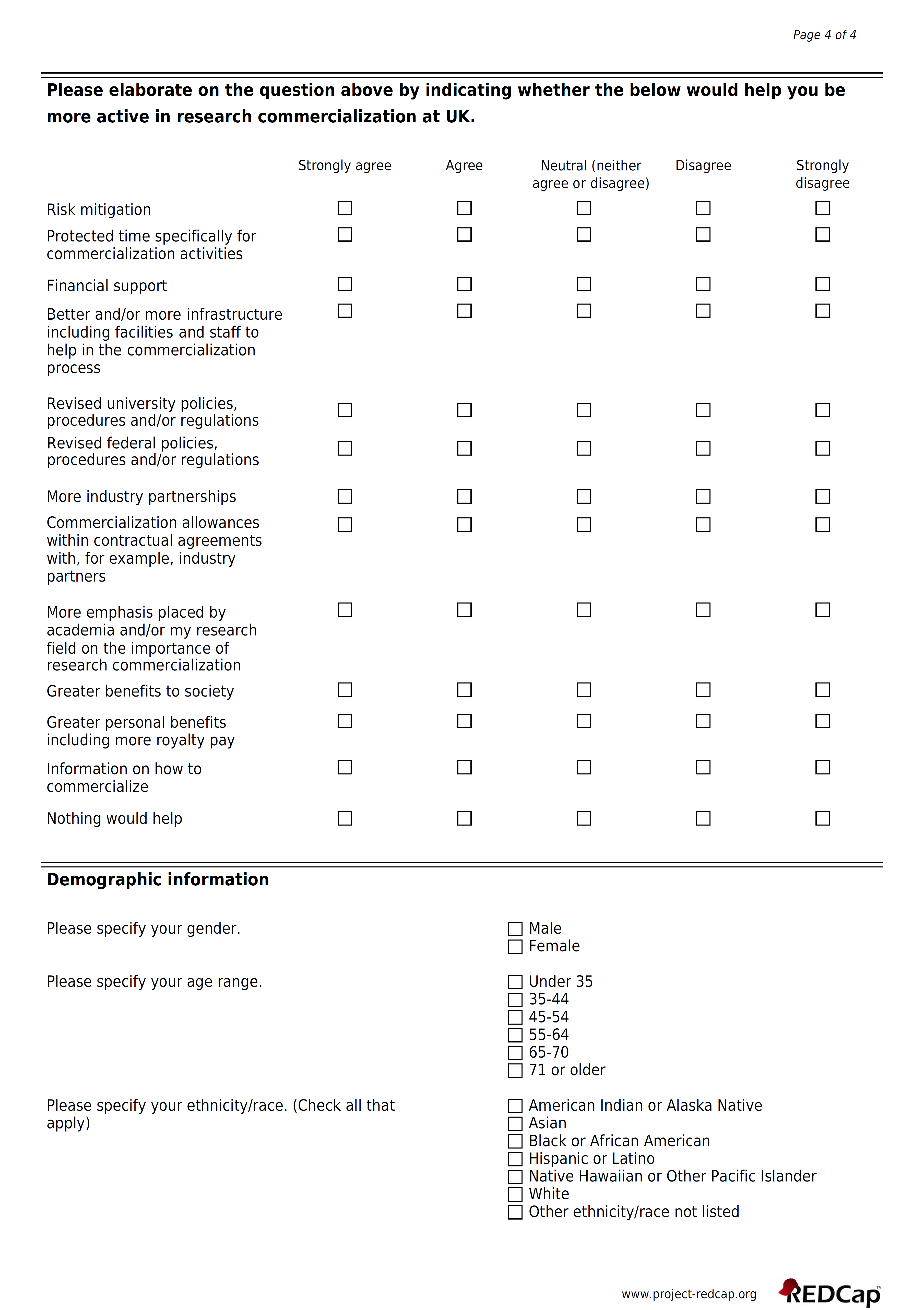

Supplement: Text S2 — (DOCX) [file pone.0072268.s013.docx]
